# Second-harmonic generation tensors from high-throughput density-functional perturbation theory

**DOI:** 10.1038/s41597-024-03590-9

**Published:** 2024-07-11

**Authors:** Victor Trinquet, Francesco Naccarato, Guillaume Brunin, Guido Petretto, Ludger Wirtz, Geoffroy Hautier, Gian-Marco Rignanese

**Affiliations:** 1 Institute of Condensed Matter and Nanoscience (IMCN), Université Catholique de Louvain, B-1348 Louvain-La-Neuve, Belgium; 2 Department of Physics and Materials Science, University of Luxembourg, L-1511 Luxembourg, Luxembourg; 3 Citrine Informatics, Redwood City, CA USA; 4 Matgenix, A6K Engineering Center, Charleroi, Belgium; 5 Thayer School of Engineering, Dartmouth College, Hanover, New Hampshire 03755 USA; 6 School of Materials Science and Engineering, Northwestern Polytechnical University, Xi’an, Shaanxi 710072 China

**Keywords:** Nonlinear optics, Computational methods, Condensed-matter physics

## Abstract

Optical materials play a key role in enabling modern optoelectronic technologies in a wide variety of domains such as the medical or the energy sector. Among them, nonlinear optical crystals are of primary importance to achieve a broader range of electromagnetic waves in the devices. However, numerous and contradicting requirements significantly limit the discovery of new potential candidates, which, in turn, hinders the technological development. In the present work, the static nonlinear susceptibility and dielectric tensor are computed via density-functional perturbation theory for a set of 579 inorganic semiconductors. The computational methodology is discussed and the provided database is described with respect to both its data distribution and its format. Several comparisons with both experimental and ab initio results from literature allow to confirm the reliability of our data. The aim of this work is to provide a relevant dataset to foster the identification of promising nonlinear optical crystals in order to motivate their subsequent experimental investigation.

## Background & Summary

Nonlinear optics has been a crucial element in optoelectronics since its inception in the 1960s^1^. Thanks to different frequency conversion phenomena, nonlinear optical (NLO) crystals allow devices to make use of the full electromagnetic (EM) spectrum despite the lack of all-solid state lasers in several frequency ranges. Consequently, this promotes the development of technologies that target specific needs depending on the wavelength of interest^2–5^. As an example, deep-UV lasers^6^ have applications ranging from pathogen detection and sterilization in medicine^7^ to photolithography in micromachining. The quantum computing field also benefits from such light sources.

NLO crystals need to meet numerous and contradicting requirements^8^. Among others, they have to 1) display a large magnitude of nonlinear optical effects, 2) possess a large laser damage threshold, 3) be transparent in the range of the application, 4) be phase-matchable (i.e., be sufficiently birefringent), and 5) be stable and synthesizable. As a consequence, it is difficult to find suitable compounds for certain spectral regions, such as the deep-UV, mid-, and far-infrared regions. The technological progress is therefore hindered in these ranges. New NLO crystals are thus highly needed.

Historically, second-harmonic generation (SHG) was the first NLO process to be discovered^9^. It consists in the conversion of an input frequency to its second harmonic, i.e., to twice its value. The propensity to this phenomenon usually serves as a good starting point to evaluate the potential of a compound to be an effective NLO crystal. Its magnitude is characterized by the nonlinear susceptibility tensor, ${\chi }_{ijk}^{(2)}(2\omega ,\omega ,\omega )$. In practice, due to the weak dispersion of ${\chi }_{ijk}^{(2)}$ far from the absorption edge, their static value (*ω* → 0) provides a reasonable approximation of its components. These can be calculated in the framework of density-functional perturbation theory^10^ (DFPT). Thanks to the increasing computational power, it is now possible to perform high-throughput calculations as demonstrated by this work.

To our knowledge, only a few collections of around a hundred experimental results exist, such as refs. ^11–13^. The publication of the latest one dates back to 2005 and reports the coefficients of only the most known materials. The authors also emphasize the existence of discrepancies between experimental values of different sources. A few computational databases exist as well in the literature such as refs. ^14–16^. However, they do not amount to more than 300 materials. Moreover, they make use of the independent-particle approximation^17^ (IPA) which does not account for the local-field effects contrarily to the perturbative approach offered by DFPT. The latter is adopted in this work to compute and make available the static nonlinear susceptibility tensor of 579 inorganic semiconductors. The electronic contribution to their static dielectric tensor is also provided since it is obtained as a side product.

The present database is made open hoping to foster the identification of promising NLO crystals and their subsequent experimental investigations. Indeed, the complexity of this type of experiments does not allow a systematic scan of existing datasets or search in the compound space, hence the need to identify outliers in computational databases. It can also serve as reference for early experimental data for both phase-matching purposes and verification of the effective magnitude of the NLO phenomenon via the tensor components or the powder SHG intensity for example. Furthermore, it can constitute a starting dataset for machine-learning projects in this field.

The outline of the current paper is as follows. To begin with, a theoretical background on nonlinear optics and the calculated properties is given. The workflow and the data format are then described in detail. The database is also represented graphically with some basic analysis of its distribution. Finally, a comparison with experimental and *ab initio* results from the literature is provided to validate the present work.

## Methods

### Theory and definitions

Under an applied electric field **E**, materials can develop a macroscopic polarization **P** such that, in general, one can write

1 $${\bf{P}}={\varepsilon }_{0}\chi {\bf{E}},$$

with the vacuum permittivity, *ε*_0_, and the susceptibility tensor, *χ*. In the framework of perturbation theory, the above equation can be expressed as a power series such that

2 $${P}_{i}={\varepsilon }_{0}{\sum }_{j}{\chi }_{ij}^{(1)}{E}_{j}+{\varepsilon }_{0}{\sum }_{jk}{\chi }_{ijk}^{(2)}{E}_{j}{E}_{k}+\,\mathrm{higher\ order\ terms}\,,$$

where the second-rank tensor, ${\chi }_{ij}^{(1)}$, is called the linear susceptibility while the third-rank tensor, ${\chi }_{ijk}^{(2)}$, is the second order (or nonlinear) susceptibility. The former is directly related to linear optics through the dielectric function (and thus through the refractive index), since

3 $${\varepsilon }_{ij}={\delta }_{ij}+{\chi }_{ij}^{(1)}.$$

The nonlinear susceptibility is responsible for nonlinear optical phenomena such as sum frequency generation (SFG) and difference frequency generation (DFG). It is important to mention that the linear and nonlinear susceptibilities are directly proportional to one another as stated by the so-called Miller’s rule^18^. The database at hand focuses on SHG, a specific case of SFG. It consists in a conversion process by which two incident photons at a frequency *ω* combine into a single one at 2*ω*. By convention, one works with the SHG tensor, i.e., a modified version of the SHG nonlinear susceptibility:

4 $${d}_{ijk}=\frac{1}{2}{\chi }_{ijk}^{(2)}.$$

It possesses in principle 27 independent components. However, this number can be reduced to 18 for physical reasons such that the Voigt notation can be adopted for the two last indices. The SHG tensor is thus often expressed in the literature as the second-order matrix *d*_*iα*_ with *i* = 1, . . . , 3 and *α* = 1, . . . , 6. By assuming a dispersionless medium, the number of independent components can then be brought down to 10. It must also be reminded that the symmetry of the crystal can bring further simplifications. This explains, for example, the absence of SHG in centrosymmetric compounds since all elements of their SHG tensor are null.

In addition to the tensor notation, a consequence of the crystalline anisotropy is the decomposition of any incident wave into two orthogonal linear polarizations at its entry into the crystal. The two allowed directions are called ordinary and extraordinary. This splitting of the wave has to be taken into account in equation (2) such that the amplitude of the nonlinear polarization can, for example, be written as

5 $$| {{\bf{P}}}^{{\rm{o}}}| ={\sum }_{i}{a}_{i}{P}_{i}^{{\rm{o}}}={\sum }_{i}{a}_{i}{\sum }_{jk}2{d}_{ijk}{E}_{j}^{{\rm{e}}}{E}_{k}^{{\rm{e}}}={\sum }_{i}{a}_{i}{\sum }_{jk}2{d}_{ijk}{b}_{j}{b}_{k}| {{\bf{E}}}^{{\rm{e}}}{| }^{2}={d}_{{\rm{eff}}}| {{\bf{E}}}^{{\rm{e}}}{| }^{2},$$

where *a*_*i*_ and *b*_*j*_ are the direction cosines of the resulting (ordinary) and incident (extraordinary) waves, respectively. Equation (5) introduces an effective coefficient, *d*_eff_, for a specific configuration of incident angles, crystal system and interaction of e- and o-waves. Its expression has been tabulated for each scenario in the literature^19,20^.

By using Maxwell’s equations in tandem with equation (5), the intensity of the generated wave can be derived such that

6 $${I}_{3}=\frac{8\,{d}_{{\rm{eff}}}^{2}\,{\omega }_{3}^{2}\,{I}_{1}\,{I}_{2}}{{n}_{1}\,{n}_{2}\,{n}_{3}\,{\varepsilon }_{0}\,{c}^{2}}{L}^{2}{{\rm{sinc}}}^{2}\left(\frac{\Delta kL}{2}\right),$$

where the numerical indices refer to the respective interacting frequencies (*ω*_3_ = 2*ω*_1_ = 2*ω*_2_ for SHG), *c* to the speed of light in vacuum, *L* to the length of the crystal, sinc to the cardinal sine function and Δ*k* = *k*_1_ + *k*_2_ − *k*_3_ to the momentum mismatch. This equation leads to three observations.

First, the resulting intensity depends on the square of the interaction length. Maximizing this factor is thus important although complex in practice.

Second, both dependence on *L*^2^ and ${d}_{{\rm{eff}}}^{2}$ are overshadowed by the squared sinc function. Indeed, it constitutes the main constraint to an efficient energy conversion process. Fortunately, this limitation can be circumvented by setting Δ*k* to 0, i.e., by achieving phase-matching (PM). Naively, this condition implies the coherent addition of the microscopic generated fields. It is a primary concern when working with NLO crystals. In the case of SHG, the PM condition can be written as

7 $$n(\omega )=n(2\omega ).$$

This is usually impossible to achieve since loss-less materials display an effect known as normal dispersion such that, far from any resonances, the refractive index is increasing with the frequency. Nevertheless, several techniques exist to bypass this phenomenon. The main focus will be on the so-called angular phase-matching (APM) due to its computational simplicity. This approach is built upon the dependence of the refractive index on the polarization and propagation direction of the wave. This optical property, also called birefringence, results from the anisotropy of the nonlinear medium. It is thus dictated by the crystal system. Consequently, the e- and o-waves do not experience the same refractive index. In the right configuration, this can lead to the PM condition, although expressed differently:

8 $${n}_{{\rm{e}}}(\omega ;{\theta }_{{\rm{m}}})={n}_{{\rm{o}}}(2\omega ),$$

where *θ*_m_ is the incidence angle for which this equation holds true. The example taken above is a type I PM, i.e., incident (generated) waves are both extraordinary (ordinary) or inversely, in a positive, i.e., *n*_e_(*ω*, *θ* = 90^°^) > *n*_o_(*ω*), uniaxial crystal, i.e., two of the three *n*_*ii*_ are equal. The interested reader can find more information in the literature regarding the convention of the incidence angles (*θ*, *ϕ*), the type II PM, negative crystals, and biaxial ones^9,21,22^. To summarize, APM can be achieved for specific combinations of incidence angles (*θ*_m_, *ϕ*_m_), type of PM, and crystal system. The database at hand also aims at helping develop the predictions of the appropriate APM configurations for its materials.

Third, the impact of the SHG magnitude on *I*_3_ is contained in the square of the effective coefficient *d*_eff_. However, since the latter depends on the crystal system and the chosen PM type, it is not trivial to quickly compare the potential of materials regarding SHG. In the literature, the Kurtz-Perry (KP) powder method^23^ offers an alternative effective coefficient. In this framework, the expression of the generated intensity resembles equation (6). The major difference comes from the substitution of *d*_eff_ by the rotational average of the SHG tensor, *d*_KP_, whose general expression reads

9 $${d}_{{\rm{KP}}}^{2}=\frac{5}{7}{\left({d}_{lmn}\right)}^{2}+\frac{19}{105}{\sum }_{i}{\left({d}_{iii}\right)}^{2}+\frac{13}{105}{\sum }_{i\ne j}\left({d}_{iii}{d}_{ijj}\right)+\frac{44}{105}{\sum }_{i\ne j}{\left({d}_{iij}\right)}^{2}+\frac{13}{105}{\sum }_{ijk,{\rm{cyclic}}}\left({d}_{iij}{d}_{jkk}\right),$$

where the subscript *lmn* can be any cyclic combination of the indices, i.e., 123, 312, and 231. The meaning of such a nonlinear coefficient has been discussed in ref. ^24^. The modification from a tensor notation to a scalar one is of fundamental importance both for an easier screening of materials databases and for their graphical representation, as it will be shown in the next sections.

Note that the above section presented only the most important equations of the theory. The interested reader can find more detail in refs. ^9,21,25^.

### Workflow

**Fig. 1:**
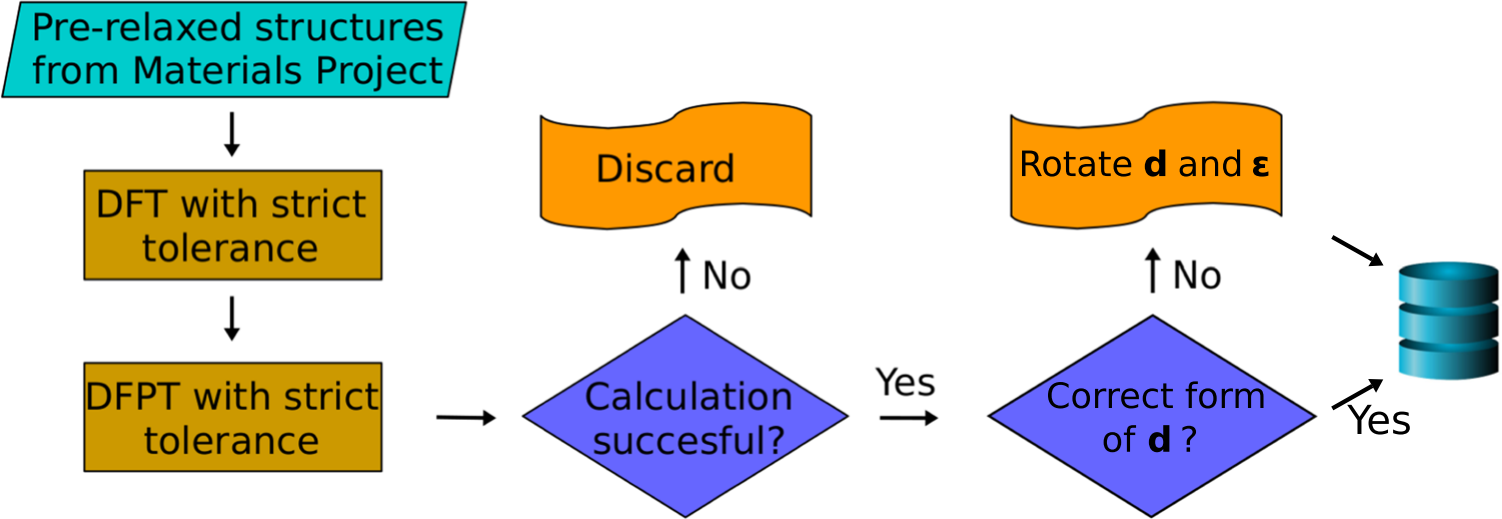
Schematic overview of the workflow implemented to compute the SHG tensor for the 579 semiconductors. The DFT and DFPT steps are detailed in Fig. 10 of ref. ^27^.

To compute the nonlinear SHG tensor, we used the open-source code ABINIT^26–28^. The high-throughput calculations were performed with the NL workflow implemented in the Abiflows package (https://github.com/abinit/abiflows). The generation of inputs and results analysis were performed using the Pymatgen^29^ and Abipy (https://github.com/abinit/abipy) python packages. The theoretical framework that allows us to compute the *d*_*ijk*_ tensor is fully described in ref. ^10^. The final database (DB) presented here contains 579 semiconductors. These materials were initially selected considering the DB from ref. ^30^, made of 4041 semiconductors. As already stated, NL phenomena at the second order are only possible in non-centrosymmetric materials. Therefore, we excluded all the centrosymmetric materials. For computational reasons related to the accuracy of the corresponding pseudopotentials, lanthanides and actinides materials were also excluded. Moreover, the calculation of the second-order susceptibility tensor requires to perform calculations with a higher accuracy than what is required for linear properties. This is difficult to achieve for magnetic systems, that have thus been discarded from our analysis. For this work, we used the pre-relaxed structures from the Materials Project^31^. The workflow implemented in Abipy and Abiflows is organized as follows. For each structure, we first perform self-consistent and non-self-consistent DFT calculations to determine the wave functions and the density. The following step consists in running DFPT simulations to obtain the second-order derivatives of the energy w.r.t. the electric field. This is basically achieved in two steps: the calculation of the derivative of the wave function w.r.t. their wave vector, and the calculation of the derivative of the first order wave function w.r.t. the applied external electric field. If the calculations are completed correctly and high accuracy is reached, the set of derivatives is then used in the last step that involves the calculation of the third order derivative w.r.t. the electric field to obtain the *d*_*ijk*_ tensor. For some space groups, the calculated SHG tensor does not show the conventional form for the corresponding point group. In those cases, both the SHG and the dielectric tensors are rotated in order to recover this conventional form. This workflow is outlined in Fig. 1. The DFT and DFPT steps are part of a more general DFPT workflow also available in the Abiflows package (https://github.com/abinit/abiflows), which allows to access all the quantities that can be computed in the framework of DFPT (see Fig. 10 of ref. ^27^). The local-density approximation (LDA) was employed to model the exchange-correlation energy^32^ and the norm-conserving pseudopotentials available in the PseudoDojo (scalar relativistic v0.3) were used^33^, along with the suggested cutoff value of each material. These pseudopotentials and cutoff have been carefully tested w.r.t. all electron codes. A reciprocal density of 3000 points per reciprocal atom was used to sample the Brillouin Zone in order to ensure the convergence while respecting the symmetry of the system. The final results were stored in the Mongo DB database engine.

## Data Records

The computed static value of the conventional SHG and electronic contribution to the dielectric tensor for a set of 579 inorganic semiconductors are publicly available in the present work. The JSON format is adopted for the processed data, which can be downloaded either from the Materials Cloud Archive^34^ (ref. ^35^) or from the Dataverse of UCLouvain (ref. ^36^). Both data records present the same format. A third record of the data is present on the MPContribs^37^ (https://contribs.materialsproject.org/projects/shg). The data is also accessible through user-friendly interactive plots (https://nlo.modl-uclouvain.org/).

### Data structure

In the Materials Cloud Archive and Dataverse of UCLouvain, the data for each compound is formatted as a JavaScript Object Notation (JSON) document^35,36^. The JSON format consists of a human-readable text commonly employed for efficiently storing and transmitting serializable data. It is based on both key/value pairs and arrays with the possibility of nested structures. Each material has its own JSON file with the top level keys described in Table 1 while the input parameters and outputs are respectively defined in Table 2 and Table 3.

**Table 1:** Table of metadata keys.

Key	Data type	Description
MP_id	string	Material id
formula	string	Chemical Formula
nsites	integer	Number of atoms in the primitive cell
crystal_system	string	Crystal system
point_group	string	Point group in Hermann-Mauguin notation
space_group	string	Space group as defined by the International Union of Crystallography
space_group_number	integer	Space group number as defined by the International Union of Crystallography
input_params	various	Input parameters used for the calculation (see Table 2)
dte_output	various	Dielecric properties computed with DFPT (see Table 3)

**Table 2:** Table of input parameter keys.

Key	Data type	Description
structure	string	Crystal structure in Crystallographic Information File (CIF) format
ecut	float	Energy cutoff (Hartree)
ngkpt	array	*k*-point grid
nshiftk	integer	Number of *k*-point grid shifts
shiftk	array	Shift of *k*-point grid
pseudopotential_md5	array	List of MD5 hashes uniquely identifying the pseudopotentials

**Table 3:** Table of output parameter keys.

Key	Data type	Description
eps_inf	array	Electronic contribution to the dielectric permittivity tensor
dte	array	Static SHG tensor (pm/V)

Table 1 is mainly comprised of metadata keys, which give a brief description and allow for the identification of the material under scrutiny. In an effort to ensure reproducible results, the input parameters used in the first-principles calculations are also provided as listed in Table 2. Table 3 details the format of the outputs, i.e., the static conventional SHG tensor in pm/V and the static high-frequency dielectric tensor. When needed, the physical units of a quantity are given in its description.

In the MPContribs record (https://contribs.materialsproject.org/projects/shg), each entry is identified by the corresponding mp-id, its formula, and a unique identifier that can be used for querying the pymatgen Structure used in the computation and other data with the MPContribs API. The main *data* header is comprised of two subheaders, *dinf* and *epsinf*, that correspond to the static conventional SHG tensor in pm/V and the static high-frequency dielectric tensor. Furthermore, they are themselves subdivided into 27 and 9 subsubheaders, respectively, to indicate each component of the respective tensors. For example, the first component of the SHG tensor is given in the *data.dinf.111* column.

### Graphical representation of the results

Usually, datasets can be described by plotting the features of interest. However, in the present case, the tensorial nature of *d*_*ijk*_ prevents any simple graphical representation. Indeed, the SHG tensor often contains more than one independent component. For the same reason, compounds with different crystal symmetry cannot be easily compared with respect to their NLO properties. Fortunately, these limitations can be circumvented by substituting an average coefficient to the SHG tensor as explained previously. In that regard, we use the KP effective coefficient, *d*_KP_ from equation (9), as it is abundantly used in the literature. This scalar quantity effectively allows for a graphical representation of the DB. It can also serve as a criterion to compare the relative SHG magnitude of materials and could thus be used in high-throughput searches for new NLO crystals. Moreover, this alternative scalar representation facilitates the investigation of “structure-property relationships” in regard to NLO phenomena.

Various plots of the database showing the relationship between the SHG response, the bandgap, and the refractive index are accessible online (https://nlo.modl-uclouvain.org/). Both the KP coefficient and static refractive index decrease with an increasing band gap. This was to be expected from Miller’s rule since *n*_*s*_ is directly related to *χ*^(1)^ via the dielectric function. The inverse relationship between *E*_*g*_ and *n*_*s*_ has been modeled in the literature as well as in a previous work involving most of us^30^. The principal feature of our data distribution is its large spread with a distinct skewness towards low KP coefficient, i.e., towards low SHG components. Although not ideal, it must be reminded that, at high bandgap, even low SHG coefficient can prove useful in practice^38,39^. From the distribution of instances among the different point groups and their respective spread of the SHG coefficient, it can be seen that the DB mostly consists of materials from the *mm*2 at 21.24% and $\bar{4}3m$ groups at 12.61%. Interestingly, the latter spans the whole range of *d*_KP_ values (up to 160 pm/V) while the former tends to have lower coefficients (up to 17 pm/V). The point groups $\bar{6}$ and 4 are particularly under-represented with 0.35% and 0.17% respectively. It is worth noting that 483 out of the 579 compounds are associated with at least one entry in the ICSD^40,41^, which comprises structures reported experimentally.

## Technical Validation

**Fig. 2:**
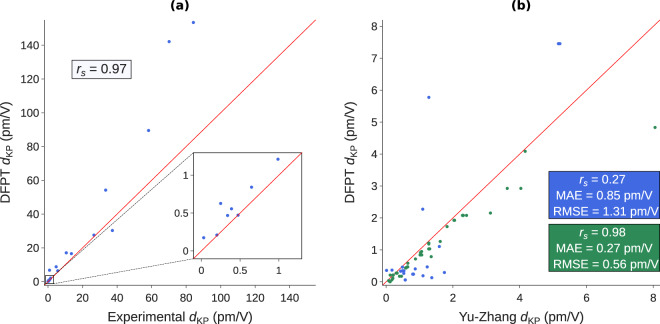
(**a**) Comparison of the KP effective coefficient between experimental values found in refs. ^11,12^ and the DFPT values of the present DB. The inset provides a closer view of the lowest values. The Spearman’s rank correlation coefficient (*r*_*s*_) is indicated to illustrate the accuracy of the ranking. (**b**) Comparison of the KP effective coefficient between theoretical values from ref. ^14^ (blue) and ref. ^15^ (green) and the DFPT values of our DB. The Spearman’s rank correlation coefficient (*r*_*s*_) as well as the mean absolute error (MAE) and root mean square error (RMSE) are indicated to illustrate the quality of the ranking.

One of the main problems in fields such as nonlinear optics is the limited amount of available experimental data. This prevents a thorough assessment of the reliability of the present database through the verification of the data accuracy. Moreover, the necessary experiments to obtain such data are all but straightforward. This often causes experimental results to disagree with each other. Nonetheless, a comparison between the DFPT data of the DB and experimental results from ref. ^11^ is presented in Table 4 for a few well-known nonlinear optical crystals. As expected from the above explanation, the error, defined as $| {d}_{i\alpha }^{{\rm{DFPT}}}-{d}_{i\alpha }^{{\rm{Exp}}.}| /{d}_{i\alpha }^{{\rm{Exp}}.}$, spans a large range. For some compounds, the theoretical components of the SHG tensor can differ by more than 200% from the experimental ones while this difference amounts to only a few percents for other materials. Due to different orders of magnitude of the SHG coefficients, this relative error cannot be considered to properly describe the accuracy of our data. Indeed, in the case of Ba(BO_2_)_2_, the DFPT value of the *d*_33_ component (0.14 pm/V) gives a good estimation of its experimental counterpart (0.04 pm/V) although the relative error reaches 250%. Furthermore, a value of 0.16 pm/V has been reported in ref. ^12^ which emphasizes the above comment on the difficulties faced by experimentalists. Thanks to the effective KP coefficient, an additional validation can be performed as illustrated by the left panel in Fig. 2. The latter consists of a parity plot that compares our DFPT results to experimental data for 19 materials^11,12^, including the ones of Table 4. Out of the 19 materials, 14 of them display a relative error between 0 and 60% approximately. It shows a qualitative agreement for most compounds, as emphasized by the inset. This observation is further supported by the high Spearman’s rank correlation coefficient (0.97). This quantity tends to 1 when both variables display the same relative ranking of their values. This supports the use of both our database and our methodology to select promising materials based on a relative ranking. The primary reason for discrepancies comes from the band gap problem inherent to DFT calculations. Indeed, the improvement of *E*_*g*_ when increasing its value with a scissor shift can lead to a decrease of the refractive index which, in turn, can lower the SHG coefficient following Miller’s rule. As a consequence, it can be stated that, in general, the present DB tends to overestimate the components of the SHG tensor. This last remark can be validated by both Table 4 and Figure 2(b).

**Table 4:** Comparison between the DFPT values from the NLO DB and experimental ones for the relevant *d*_*iα*_ components of some well-known nonlinear optical crystals as collected in ref. ^11^.

Formula	MP-id	Point group	$| {{\boldsymbol{d}}}_{{\boldsymbol{i}}{\boldsymbol{\alpha }}}^{{\bf{Exp}}{\boldsymbol{}}.}|$ (pm/V)	$| {{\boldsymbol{d}}}_{{\boldsymbol{i}}{\boldsymbol{\alpha }}}^{{\bf{DFPT}}}|$ (pm/V)	$| {{\boldsymbol{d}}}_{{\boldsymbol{i}}{\boldsymbol{\alpha }}}^{{\bf{DFPT}}}-{{\boldsymbol{d}}}_{{\boldsymbol{i}}{\boldsymbol{\alpha }}}^{{\bf{Exp}}.}| /{{\boldsymbol{d}}}_{{\boldsymbol{i}}{\boldsymbol{\alpha }}}^{{\bf{Exp}}.}$
Ba(BO_2_)_2_	mp-5730	3*m*	*d*_22_(1.064 *μ*m) = 2.20	*d*_22_ = 3.01	36.32%
			*d*_15_(1.064 *μ*m) = 0.04	*d*_15_ = 0.05	25.00%
			*d*_33_(1.064 *μ*m) = 0.04	*d*_33_ = 0.14	250.00%
LiB_3_O_5_	mp-3660	*mm*2	*d*_31_(1.0642 *μ*m) = 0.67	*d*_31_ = 0.88	31.34%
			*d*_32_(1.0642 *μ*m) = 0.85	*d*_32_ = 1.10	29.41%
			*d*_33_(1.0642 *μ*m) = 0.04	*d*_33_ = 0.12	200.00%
LiNbO_3_	mp-3731	3*m*	*d*_22_(1.064 *μ*m) = 2.10	*d*_22_ = 1.72	18.10%
			*d*_15_(1.064 *μ*m) = 4.35	*d*_15_ = 8.66	99.08%
			*d*_33_(1.064 *μ*m) = 27.20	*d*_33_ = 28.19	3.64%
KTiPO_5_	mp-6268	*mm*2	*d*_31_(1.313 *μ*m) = 1.40	*d*_31_ = 1.72	22.86%
			*d*_32_(1.313 *μ*m) = 2.60	*d*_32_ = 3.68	41.54%
			*d*_33_(1.313 *μ*m) = 11.1	*d*_33_ = 11.87	6.94%
GaAgS_2_	mp-5342	$\bar{4}2m$	*d*_36_(10.6 *μ*m) = 12.50	*d*_36_ = 20.16	61.28%
GaAgSe_2_	mp-5518	$\bar{4}2m$	*d*_36_(10.591 *μ*m) = 39.50	*d*_36_ = 63.86	61.67%
ZnGeP_2_	mp-4524	$\bar{4}2m$	*d*_36_(10.591 *μ*m) = 68.90	*d*_36_ = 105.90	53.70%

The right panel of Figure 2 provides a comparison of our database with two datasets^14,15^ built from first-principles. Contrarily to the present work, they used an IPA approach instead of DFPT. It can be seen from the blue markers that our data does not display the same relative ranking as ref. ^14^ as emphasized by the low Spearman coefficient (0.27). However, it must be noted that the orders of magnitude of the SHG coefficient are still predicted in an appropriate manner. On the other hand, our SHG coefficients agree very well with the ones of ref. ^15^ as depicted by the green markers. The Spearman coefficient is 0.98 which indicates that the relative ranking stays the same in both datasets. Although it is difficult to identify a specific reason for the discrepancies, the difference in crystalline structure due to different optimization criteria between the Materials Project and refs. ^14,15^ is surely a factor since NLO properties highly depend on the geometry. As an example, it has already been observed that a difference in the lattice parameters of less than 1% can result in a variation of the SHG response by more than 5%^42^.

This rather strong dependence of the SHG results on the input structure is important for justifying the choice made for the present dataset. The structures were indeed relaxed using the Perdew-Burke-Ernzerhof (PBE) generalized-gradient approximation^43^ for the exchange-correlation energy instead of the LDA. In fact, neither LDA nor PBE perfectly reproduces the experimental structures, so that there is no reason to favor one over the other. Unfortunately, there is no systematic trend in the difference between the SHG responses computed using the experimental and theoretical structures. Additional tests were conducted by computing the SHG response of a few LDA-relaxed structures. Although the lattice parameters were constantly decreased by 1% to 7%, the KP coefficient of the SHG tensor increased or decreased without any straightforward trend. The magnitude of this change was also unpredictable, i.e., from a few percents up to 100%, especially for small values of the coefficient. The relative ranking of the test set did not change, but abnormalities cannot be excluded.

Recently, Xie *et al*. reported that PNO can be considered a new promising deep-UV NLO crystal^44^. It adopts the space group *I*2_1_2_1_2_1_ and is conventionally represented in an orthorhombic cell. They report a value of 2.33 pm/V for its *d*_36_ component. This compound is actually part of the present database and its *d*_36_ element is predicted to be 3.41 pm/V. The overestimation can again be explained by the bandgap problem since Xie *et al*. used an HSE bandgap. Since PNO is located in the interesting region of our map, i.e., the high *d*_KP_/high *E*_*g*_ front, this illustrates the possible use of our DB to identify promising optical materials.

As introduced in the previous sections, to be exploitable in practical devices, nonlinear optical crystals must be able to achieve phase-matching conditions in order to realize an efficient energy conversion. For this reason, the predicted SHG tensor of the DB can be used to theoretically determine *d*_eff_, as defined in equation (5), for all combinations of incidence angles (*θ*, *ϕ*), and more specifically for the ones verifying the PM condition. The results of such approach are described in the following for a material from our DB, KTP (KTiPO_5_), a widely investigated NLO material. Experimental data are also used to assess the validity of the ab initio values in this framework.

**Fig. 3:**
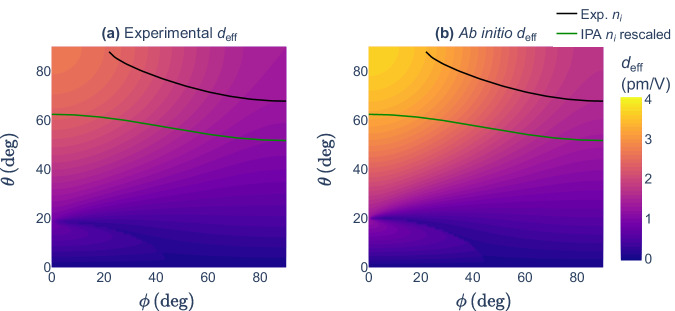
Type II phase matching for SHG in KTP at the fundamental wavelength *λ* = 1064 nm. The solid curves correspond to the phase-matching combinations of the incidence angles (*θ*, *ϕ*) as derived in ref. ^46^. The black and green curves refer to the use of experimental^46^ and ab initio (Optic utility of ABINIT) refractive indices rescaled on static DFPT results (see text), respectively. The colorbar indicates the value of *d*_eff_ (see equation (5)) for each (*θ*, *ϕ*) as analytically derived in ref. ^20^. The effective coefficient was computed from an experimental^48^ SHG *d*_*ijk*_ (left) and an ab initio one via static DFPT from the present DB (right).

Figure 3 displays a contour plot of *d*_eff_ with respect to the incidence angles for a type II PM. The experimental (left panel) and the DFPT effective coefficients (right panel) show a good qualitative agreement regarding both their trends and their magnitude. As explained previously, the characteristic overestimation due to the bandgap problem can be observed. The solid curves indicate the incidence angles for which the PM condition is verified, i.e., (*θ*_m_, *ϕ*_m_). In order to determine these directions, the refractive indices at the fundamental and second-harmonic frequency were computed in the IPA as implemented in the Optic utility of ABINIT. A scissor shift of the bandgap was performed to match the experimental value^45^. Since they constitute a slowly increasing function in this range of frequencies, the refractive indices were then rescaled thanks to the following factor: ${n}_{i}^{{\rm{DFPT}}}/{n}_{i}^{{\rm{IPA}}}(\omega \to 0)$, where the superscript DFPT corresponds here to a DFPT calculation with a scissor shift of the bandgap. This effectively allows ${n}_{i}^{{\rm{IPA}}}(\omega )$ and ${n}_{i}^{{\rm{IPA}}}(2\omega )$ to fall closer to their experimental counterpart. The resulting theoretical PM loci do not agree very well with the ones based on experimental *n*_*i*_. This discrepancy is primarily caused by the difference in refractive indices. As stated in ref. ^46^, changes in the third decimal place can lead to considerable modifications of the resulting PM angles. When the refractive indices are not rescaled, the discrepancy is thus even more severe.

**Fig. 4:**
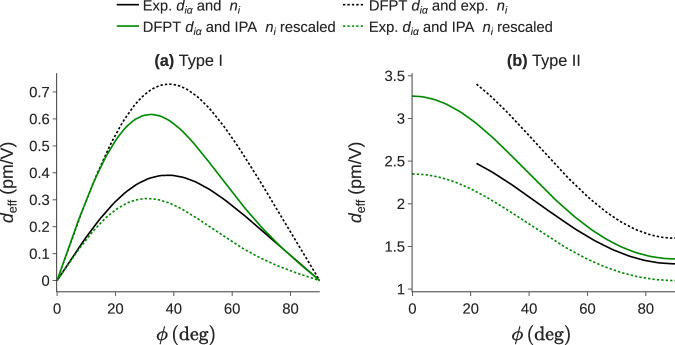
Nonlinear effective coefficient *d*_eff_ as a function of the incidence angle *ϕ* and its corresponding phase-matched incidence angle *θ*_m_ in the case of KTP^20,46^. The different lines correspond to: (i) Continuous black: fully experimental results; (ii) Dashed black: experimental refractive indices^46^ with theoretical SHG tensor; (iii) Continuous green: fully DFPT predictions; (iv) Dashed green: DFPT refractive indices with experimental *d*_*iα*_^48^.

In Fig. 4, the effective coefficient is plotted as a function of the angle *ϕ* when PM is achieved. Both type I and type II PM are covered. The latter can be directly related to the solid curves of Fig. 3. Four different configurations are compared: fully experimental, fully theoretical, and two mixes of experimental and theoretical *d*_*iα*_ and *n*_*i*_. Indeed, the use of experimental refractive indices in tandem with DFPT SHG tensors allows to recover the correct PM trend with an over-estimation of the effective coefficient as emphasized before. The trends and the order of magnitude of *d*_eff_ are similar for each case. In conclusion, the present DB can effectively be used as a first approach to predict the effective coefficient in PM conditions.

## Usage Notes

The present DB is addressed to any researcher or engineer in search of new NLO materials. A wide variety of applications can be targeted such as in the medical domain, in the energy one, or in the communications sector through the development of versatile optoelectronic devices in need of frequency conversion phenomena. Moreover, current NLO crystals could be replaced to increase efficiency or solve environmental concerns. For the sake of accessibility, the interested reader can interact with different visualizations of the present data online (https://nlo.modl-uclouvain.org/). Please note that the choice of browser can affect the rendering of the figures. This provides an intuitive and fast way to screen the present DB and access the relevant properties. Clickable markers were implemented to direct the user towards the Materials Project website. This feature enables the user to gain more insights into the materials through other characteristics such as, e.g., the stability, the birefringence, or the density. In addition, this DB paves the way to a broader use of machine learning and data-mining techniques in the field of nonlinear optics. It creates opportunities to identify key structural or chemical features at play while accelerating the discovery of NLO crystals through accurate and time-efficient machine-learned model. We plan to continually expand the database by computing new materials.

## Data Availability

The open source code ABINIT^26–28^ is used throughout this work for calculating the optical properties. ABINIT is distributed under the GNU General Public Licence. The workflows used to run the simulations are implemented using FireWorks (https://github.com/materialsproject/fireworks) as workflow manager^47^ and specific workflows are available in the Abiflows package (https://github.com/abinit/abiflows). The Pymatgen^29^ and Abipy (https://github.com/abinit/abipy) python packages are used to generate inputs and analyze the results. Pymatgen is released under the MIT (Massachusetts Institute of Technology) License and is open source. AbiPy is released under the GNU GPL license. FireWorks is released under a modified BSD license.
